# SARS Molecular Detection External Quality Assurance

**DOI:** 10.3201/eid1012.040416

**Published:** 2004-12

**Authors:** Christian Drosten, Hans Wilhelm Doerr, Wilina Lim, Klaus Stöhr, Matthias Niedrig

**Affiliations:** * Bernhard Nocht Institute for Tropical Medicine, Hamburg, Germany;; † University of Frankfurt, Frankfurt, Germany;; ‡ Government Virus Unit Hong Kong, Hong Kong, China;; § World Health Organization, Geneva, Switzerland;; ¶ Robert Koch Institute, Berlin, Germany

**Keywords:** Quality assurance, NAT, PCR, SARS, WHO, dispatch

## Abstract

Inactivated severe acute respiratory syndrome–associated coronavirus samples were used for an external quality assurance study within the World Health Organization SARS Reference and Verification Network and other reference institutions. Of 58 participants, 51 correctly detected virus in all samples >9,400 RNA copies per milliliter and none in negative samples. Commercial test kits significantly improved the outcome.

Severe acute respiratory syndrome (SARS) is an infectious interstitial pneumonia that causes death in a considerable portion of patients. The first epidemic of SARS began in November 2002 in southern China, spread to all five continents, and was interrupted in July 2003. It caused 774 deaths among the 8,098 cases. Two laboratory-associated infections and four new isolated cases have since occurred (*1*). SARS is caused by a novel coronavirus (SARS-CoV) that is shed in patients' respiratory secretions after infection (*2**–**5*). Immune response to SARS-CoV appears with a latency of up to 4 weeks from infection, and the concentration of virus particles varies greatly between patients or types of clinical samples. Thus achieving a reliable virologic diagnosis early after disease onset is difficult. Highly sensitive methods for virus detection, such as reverse transcription–polymerase chain reaction (RT-PCR) are required to confirm SARS in the acute phase and prevent transmission.

Molecular detection methods have been developed by several research laboratories, and the first commercial test kits have become available (*6**,**7*). The performance of such tests, however, has only been evaluated in pilot feasibility studies. Little data exist about the relative performance of different laboratories and methods. The World Health Organization (WHO) has made the comparing and standardizing of laboratory tests an issue of high priority in SARS research (*8*). Comparative testing of characterized samples is a direct way to identify weaknesses of single laboratories or certain methods.

## The Study

We present the results of the first external quality assurance study on SARS-CoV molecular detection. Ninety-three institutions involved in laboratory diagnostics of SARS were invited to participate in the study. Invitees were members of the international WHO SARS Reference and Verification Laboratory Network (*9*) or national and regional SARS reference laboratories. The study was announced as an external quality assurance study on diagnostic proficiency, which included certifying and publishing the results in a comparative and anonymous manner. Fifty-eight laboratories from 38 countries (21 European, 9 Austral-Asian, 7 North and South American, and 1 African) eventually enrolled in the study.^1^ Four companies that produced commercial diagnostic test systems also participated but were evaluated separately because they do not fulfill public health duties.

Virus material was obtained from supernatants of Vero cell cultures collected one day after infection with SARS-CoV strains Frankfurt 1 and HKU-1. The supernatants were heated to 56°C for 1 h and γ irradiated with 30 kGy. Residual infectivity was excluded by Vero cell cultures (3 passages). Aliquots of the inactivated virus stock solutions were lyophilized and redissolved, and the virus RNA was quantified by two different noncommercial real-time RT-PCR assays (*2**,**6*). Virion integrity was confirmed by morphology by electron microscopy (data not shown). Test samples for the study were generated by diluting the inactivated virus stock solutions in human fresh-frozen plasma testing negative for HIV-1, hepatitis B virus, hepatitis C virus, and SARS-CoV by RT-PCR. Aliquots of 100 µL each were then lyophilized and shipped at ambient temperature to the participating laboratories. Each participant received a coded panel of seven positive and three negative samples. Virus-positive samples contained 94–940,000 RNA copies per milliliter after resuspending in 100 µL of water. The participants were asked to analyze the material with the molecular methods they routinely use in suspected cases in humans. Details about the methods were requested, such as the sources of RT-PCR primers and protocols, the type of extraction method used, and suppliers and types of commercial kits, if used. The following two criteria were chosen as minimum requirements for overall proficiency. First, laboratories had to correctly detect the four samples containing >9,400 copies of viral RNA per milliliter, a concentration well above the detection limit of published and commercial nucleic acid amplification tests (NAT) for SARS-CoV (*6**,**7**,**10**–**12*). Second, no false-positive results were allowed with the negative samples. Indeterminate results in positive samples were treated as negative and in negative samples were treated as positive since the application of NAT usually does not involve indeterminate endpoints, and laboratories should be able to resolve unclear results by double testing with another amplification assay (*13*).

Before evaluating the performance of individual laboratories, we determined how many participants managed to detect virus in each sample (Table 1). The concentration-dependent, cumulative positivity rates per sample corresponded exactly with the response rates calculated by a probit regression analysis, which is equivalent to a dose-response model (Figure, p < 0.0001). The model could predict for the average laboratory that 50% of all test results could be expected to be correctly positive when 158 (95% confidence interval [CI] 76.55–269.15) copies of virus RNA per milliliter of sample were present, and 95% with more than 11,220 (95% CI 5,675–31,988) copies per milliliter. Good compliance with the model furthermore confirmed that all samples contained the expected concentration of RNA upon reception by the participants and that no RNA degradation had occurred even in samples containing low amounts of virus.

**Table 1:** Positive samples in test panel^a^

Sample code	SARS-CoV strain	Virus RNA concentration copies/mL	Fraction of laboratories with positive detection (%)
S-CV2	Frankfurt 1	940,000	100
S-CV9	Frankfurt 1	94,000	98.3
S-CV6	HKU-1	23,500	98.3
S-CV4	Frankfurt 1	9,400	94.8
S-CV10	HKU-1	2,350	87.9
S-CV1	Frankfurt 1	940	70.7
S-CV5	Frankfurt 1	94	43.1

^a^SARS-CoV, severe acute respiratory syndrome–associated coronavirus.

**Figure:**
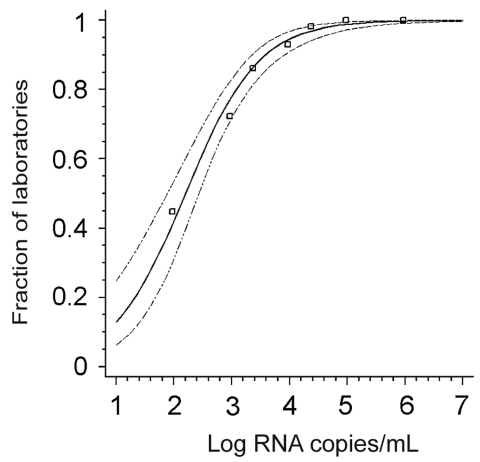
Probit analysis of the fractions of laboratories achieving a positive result (y-axis) in relation to the virus RNA concentration in a given positive sample (x-axis). Data points represent individual samples in proficiency test panel. The thick line is the regression line calculated on the basis of a probit model (dose-response curve); the thin lines are 95% confidence intervals. Data fit into the model with p < 0.0001.

Applying the proficiency criteria, 51 (88%) of 58 laboratories passed the minimum requirements for successful participation. Failure in three laboratories was due to lack of sensitivity, in three due to false-positive results, and in one due to both. Thirteen of 51 successful laboratories (22.4% of all 58 participants) could also detect the virus in all three weakly positive samples (<2,350 copies/mL), and another 17 missed only one positive sample. Ten of the 58 laboratories issued indeterminate results in one or more samples.

Whether common technical factors would influence the performance of laboratories was also assessed. We subjected cumulative results from low concentration samples (<2,300 copies/mL) to analysis of variance (ANOVA) analysis. The overall positivity rate in these samples was 65.6% (95% CI 56.1%–75.0%). Seven technical factors (Table 2) were used to characterize the test procedures each laboratory was using. Only use of commercial RT-PCR test kits made a significant difference with regard to total sensitivity. This finding was in concordance with results of the four participating companies who manufacture these kits: all were 100% correct. Fourteen of 58 participants used commercial test kits. For noncommercial tests, whether laboratories developed primers themselves or adapted from other researchers did not make a difference. This finding might be due to availability of well-evaluated primers through a WHO internet resource during the outbreak (*14*). Forty-two of the 58 participants used at least one procedure listed on this site.

**Table 2:** Factors influencing the performance of laboratories^a^

Possible technical influence factors	No. of laboratories	Positive influence on sensitivity p value
Qiagen viral RNA extraction kit	38	0.9
Roche MagnaPure/HighPure extraction kit	7	0.2
Silica particle-based extraction method (Boom)	9	0.9
Primers originally developed in own laboratory	16	0.5
Any nested PCR assay	25	0.9
Any real-time PCR assay	37	0.7
Any commercial test kit	14	0.03

^a^Analysis of Variance (ANOVA) by factor, eliminating the influence of other factors; PCR, polymerase chain reaction.

We finally assessed whether laboratories belonging to the international WHO SARS Reference and Verification Network (*9*) were more proficient in SARS molecular detection than others. In the three samples containing <2,350 copies of SARS-CoV RNA per milliliter, the network laboratories achieved a cumulative fraction of correct positive results of 79.5% (95% CI 60.2%–98.9%) as opposed to 61.5% (95% CI 50.6%–72.4%) in the other labs participating in the study. This difference was not significant (p value = 0.11, *t*-test).

## Conclusions

The results of this first external quality assurance study on SARS-CoV molecular detection are assuring. Compared to an earlier study on molecular testing for filoviruses, Lassa virus, and orthopoxviruses, using very similar proficiency criteria (*15*), almost double the portion of participating laboratories completed the study successfully (88% vs. 45.8%). On the other hand, this study only examined paramount issues like sensitivity and control of contamination. Validation of other aspects, like cross-reactivity of primers or control of PCR inhibition, is the responsibility of each diagnostic laboratory.

Commercial tests clearly were the preferred way of achieving good diagnostic performance, possibly because SARS-CoV is a pathogen with which relatively few laboratories have had experience. However, developing and approving commercial tests is a lengthy process and high costs limit their application. Other approaches have to be adopted for efficiently providing good diagnostic tools in immediate response to an infectious disease outbreak. WHO's strategy of disseminating essential information through a public Internet resource before publication has proven successful. Laboratories have willingly shared protocols and positive control material with other institutions, enabling qualified diagnostics within weeks after the primary description of the new virus. The benefit is proven by good overall results in this study.

International strain collections should be complemented with noninfectious reference material of rare pathogens. Until now, such material has been available only for highly prevalent agents like HIV-1, herpes viruses, or hepatitis viruses. For SARS-CoV, reference material has been created in this study for the first time. All samples described can be obtained for a nonprofit charge through the WHO SARS Reference and Verification Laboratory Network.

## Footnotes

1 University Vienna, Vienna, Austria; University Hospital Leuven, Leuven, Belgium; Statens Serum Institut, Copenhagen, Denmark; Health Protection Agency, London and Salibury. England; University of Helsinki, Helsinki, Finland; Institut Pasteur, Paris, France; Bernhard Nocht Institut, Hamburg, Germany; Philipps Universität, Marburg, Germany; Robert Koch-Institut, Berlin, Germany; University Frankfurt, Frankfurt, Germany; M & LAT, Berlin, Germany; Artus GmbH, Hamburg, Germany; Euroimmun, Lübeck, Germany; Aristotelian University, Thessaloniki, Greece; University of Athens, Athens, Greece;National Center for Epidemiology, Budapest, Hungary; University Hospital Reykjavik, Reykjavik, Iceland; Chaim Sheba Medical Center, Tel Hashomer, Israel; Army Medical and Veterinary Research Center, Rome, Italy; Istituto Nazionale Malattie Infettive, Rome, Italy; Istituto Superiore di Sanità, Rome, Italy; Erasmus MC, Rotterdam, the Netherlands; RIVM, Bilthoven, the Netherlands; Leiden University Medical Center, Leiden, the Netherlands; Norwegian Institute of Public Health, Oslo, Norway; National Institute of Health, Warsaw, Poland; National Institute of Health, Lisboa, Portugal; Central Research Institute for Epidemiology, Moscow, Russia; University of Ljubljana, Ljubljana, Slovenia; Instituto de Salud Carlos III, Madrid, Spain; Swedish Institute for Infectious Disease Control, Solna, Sweden; IKMI, St. Gallen, Switzerland; Hôpital Central Universitare Geneve, Geneva; Switzerland; Institut Pasteur de Dakar, Dakar, Senegal; National Institute of Health, Buenos Aires, Argentina; Fundacao Oswaldo Cruz, Rio de Janeiro, Brazil; Canadian Center for Human & Animal Health, Winipeg, Canada; Sunnybrook & Women's College Health Sciences Centre, Toronto, Canada; Clinical and Central Public Health Laboratory, Etobicoke, Canada; National Institute of Health, Nunoa Santiago, Chili; Instituto Conmemorativa Gorgas, Panama, Panama; CDC, Atlanta, Georgia, USA; Focus Technologies, Cypress, CA, USA; Governmental Virus Unit, Hong Kong, China; University of Hong Kong, Hong Kong, China; Peking University, Peking, China; CDC Beijing, Beijing, China; NIHRD, Jakarta, Indonesia; National Institute of Infectious Diseases, Tokyo, Japan; National Institute of Health, Seoul, Korea; Singapore General Hospital, Singapore; National Institute of Health, Nonthaburi, Thailand; Westmead Hospital, Westmead, Australia; Victorian Infectious Disease. Laboratory, Melbourne, Australia; LabPlus, Aukland Hospital, Aukland, New Zealand; Institute of Environmental Research, Porirua, New Zealand.
